# The role of fathers in overweight prevention: an analysis of a Caribbean cohort

**DOI:** 10.1017/gheg.2018.12

**Published:** 2018-08-28

**Authors:** J. A. Smith, K. D. Rocke, S. M. Charles, S. M. Chang, A. S. Wright, S. P. Walker, E. M. Taveras, M. K. Tulloch-Reid

**Affiliations:** 1 Epidemiology Research Unit, Caribbean Institute for Health Research, The University of the West Indies, Mona, Kingston, Jamaica; 2 Environmental Health Sciences, Department of Epidemiology and Biostatistics, College of Human Medicine, East Lansing, MI, USA; 3 Georgia Southern University, Georgia, USA; 4 Division of General Academic Pediatrics, Department of Pediatrics, Massachusetts General Hospital, Boston, MA, USA

**Keywords:** Caribbean, early childhood, overweight prevention, role of fathers

## Abstract

**Background:**

Family-based strategies to reduce the risk of overweight in childhood are needed in the Caribbean.

**Aim:**

To investigate the associations between parental characteristics and risk of overweight and explore possible mechanisms.

**Methods:**

Data from a parenting intervention were analysed. Parental characteristics were obtained by questionnaire at enrolment. At 18 months, 501 infants (82.9% of cohort) had weight and length measured using standardized methods. The association of parents’ characteristics with risk of infant overweight was assessed using random-effects logistic regression. Four focus groups among mothers in Jamaica were conducted to explore mechanisms.

**Results:**

Overall, 20.6% of infants were ‘at risk of overweight’. Fathers were present in 52% of households. Fathers’ presence [OR (95% CI) 0.60 (0.37–0.96)] was associated with reduced risk of overweight independent of socioeconomic status. Mothers reported that fathers encouraged healthier practices.

**Conclusion:**

Fathers may be important agents of change in intervention strategies to prevent childhood overweight.

## Introduction

It is estimated that the global prevalence of childhood overweight and obesity increased from 4.2% (26.9 million children <5years) to 6.7% (42.8 million <5years) during the period 1990–2010 [1]. Estimates in 2010 suggested that 92 million children worldwide under 5 years were at risk of being overweight if there was no change in current trends [1]. Analyses conducted by Ng *et al.* estimated the global prevalence of overweight and obesity in children (<18 years old) to be 13.4% in boys and 19.9% in girls [2]. In low- and middle-income countries, 32 million children under 5 years are estimated to be overweight [3].

Lifestyle behaviours, which begin in childhood, can result in overweight and obesity with parental characteristics and practices having a major influence on the development of these behaviours in young children [4]. Studies conducted in developed countries have investigated the influence of the family environment, including the socioeconomic status (SES) of the family and the marital status of the mother on the risk of overweight during early childhood [5–7]. Hawkins *et al*. reported that children of families where the mother was the sole provider were at increased risk of overweight, compared with families where both parents were present [8]. Reduced risk of overweight in young children was also demonstrated when there was a male partner or spouse in the home [9].

There is a growing prevalence of childhood overweight and obesity in the Caribbean region with heightened concern at the regional and national level [10]. In 2012, overweight prevalence in children under 5 years old in Jamaica was 7.8% [11]. National survey data from 2012 indicate that fathers were present in 41.4% of households in Jamaica with more children in the high-income group having the father present in the home than those in lower income groups [11].

Despite the growing concern about childhood overweight, little is known about the impact of parental factors on overweight in early childhood in the region. We therefore investigated the role of parent characteristics, particularly paternal characteristics, on childhood overweight in participants involved in a multi-island parenting intervention. We also explored mothers’ perceptions of influences on child weight to identify the factors that might be useful in developing interventions to prevent overweight in Caribbean children.

## Methods

### Study design

A sequential mixed-methods design was utilized to explore the risk factors for overweight in early childhood.

#### Quantitative study

Data from a multi-island cluster randomized controlled trial of a parenting intervention delivered through child health clinics were used to examine the associations between parent characteristics and child weight status [12]. The parenting intervention demonstrated responsive play techniques to mothers during their usual child health clinic visits by community health staff.

### Ethical approval

The study protocol was approved by the Ethics Committees of the University of the West Indies and the Ministries of Health, Jamaica, Antigua and St. Lucia.

### Participants

Six hundred and one mother and infant dyads were recruited at the 6–8-week postnatal visit from 39 child health centres, in Jamaica (*n* = 20 centres), St. Lucia (*n* = 9) and Antigua (*n* = 10) between August 2011 and March 2012. Infants were excluded if they were born very preterm (<35 weeks), were from a multiple birth, had stayed in special care/hospital nursery >48 h post-delivery, were in day care or had no consistent caregiver, or if the person with the infant at recruitment was not the mother or primary caregiver. Written informed consent was obtained from each mother prior to participation. At the 18-month visit, 501 (82.9%) mother and infant dyads were reassessed.

### Measurements

Questionnaires were administered to mothers at the time of recruitment by interviewers trained by the research team. Information on age, highest school grade completed, maternal and paternal occupation and presence of father in the home was obtained. SES was assessed using information on sanitation, possessions and crowding. The Center for Epidemiologic Studies Depression Scale (CES-D) [13] questionnaire was administered to identify maternal depressive symptoms, and the Peabody Picture Vocabulary Test-IV (PPVT-IV) [14] was administered to assess the mothers’ receptive vocabulary. Further details of methods and reliability have been reported previously [12]. Infant birth weight and length were obtained from child health records. At the 18-month visit, infant weight, length and head circumference were measured utilizing standard procedures.

#### Qualitative study

### Participants

To explore maternal perceptions of risk factors for overweight, 30 mothers of infants aged 6–24 months, attending two urban and two rural child health clinics in Jamaica were recruited to participate in four focus group discussions. The aim was to improve our understanding of family experiences in early life that may influence the risk for overweight in the Jamaican context. Recruitment took place from November 2013 to January 2014 with the assistance of community health workers at the clinics. Similar exclusion criteria used for the mothers in the parenting intervention study were applied. Written informed consent was obtained from each mother prior to participation.

### Ethical approval

The study protocol was approved by the Ethics Committee of the University of the West Indies.

### Data collection

Focus group discussions were moderated by a trained facilitator with the two lead authors (J.A.S and K.D.R) as note takers. The facilitator and note takers had no prior relationship with any of the participants. A semi-structured focus group guide from the Family Experience in Early Life Study [15] was used to guide the focus group sessions. The questions in the focus group guide were piloted by one of the researchers in the team (J.A.S) with nine mothers of infants <24 months who attended two other urban specialist clinics. The pilot was utilized to assess comprehension of the questions and the ability to gather the information needed to assess the risk factors for overweight in the Jamaican setting. No modifications of the focus group guide questions were needed.

The discussion guide domains included risk factors for overweight and obesity in childhood based on previous research. Themes included beliefs and behaviours surrounding snacking, beliefs about childhood obesity and their experiences and perceptions of social, community and interpersonal support that influence the health of the child. Audio recordings utilizing two digital recorders and written field notes were obtained. Focus group discussions were held for approximately 120 min. We offered US$10 as a compensation to all mothers to cover the cost for transportation to the focus group location. Child care services were also provided during the focus group discussions.

### Analysis

Body mass index (BMI) *z*-scores for each infant were calculated from the weight and length measurements collected at the 18-month visit using the WHO Anthro Program (V3.1.0). Children with a BMI *z*-score of >1 were classified as ‘at risk of overweight’ [16]. Parental and infant characteristics are presented using descriptive statistics including means, standard deviations and proportions. Comparisons by country for the participant characteristics were conducted using χ^2^ and Student *t* tests. Random-effects logistic regression models taking recruitment clinic into account were used to identify the characteristics associated with the risk of overweight. In model 1, child birth weight, maternal characteristics [age, education, occupation, receptive vocabulary (PPVT), depressive symptoms score and SES] were entered, and in model 2, child birth weight, paternal characteristics (occupation and presence in the home) and SES were entered. Model 3 included child birth weight, SES and the maternal and paternal characteristics associated with child risk of overweight in the first two models. These were maternal depressive symptoms, paternal occupation and presence in the home. Intervention group assignment from the parenting study was included as a dummy variable in all models. We also calculated intraclass correlation coefficients to explore the effect of country on any associations. An SES score was derived by factor analysis using the number of household possessions out of 14, crowding in the home (persons per room) and ratings of water supply and toilet facilities [17]. Data were analysed using STATA version 12 and 14.

For the qualitative study, data from audio recordings were transcribed verbatim utilizing a transcription service and supported with field notes, which were integrated into the analysis. Using immersion and crystallization techniques [18], four research team members independently examined the content of the transcripts repeatedly for familiarity. Ideas and concepts relevant to the study objectives were identified and categorized into themes through an iterative process. The team members then assembled to compare and discuss the themes that emerged, which included the intensity and specificity of responses. Major emergent themes around risk factors for childhood overweight and maternal perceptions of fathers’ role in risk factors for overweight were identified through an iterative process. For these analyses, only those themes relevant to the influence of the father in the home will be discussed. One limitation of the study is that a pre-determined number of focus groups were conducted. As such, the conclusions of the study are moderated since theoretical saturation may not have been attained.

## Results

### Quantitative

Five hundred and one infants (51.7% males and 48.3% females) had measurements conducted at the 18-month visit. Of these, 327 were from Jamaica, 83 from St. Lucia and 91 from Antigua. Jamaican mothers who participated in the follow-up were significantly older (*p* = 0.01) than those who did not. There were no other significant differences in maternal or paternal characteristics, birth weight or infant gender between those who participated in the follow-up and those who did not (data not shown). A higher percentage of mothers (47.3%) who participated in the follow-up were in the 15–24 years old age group (Table 1). Eighty-four per cent of mothers had secondary school education, and there were no significant differences in education level by country (Table 1). Forty-three per cent of mothers were in semi-skilled occupations with Jamaican mothers having the highest proportion of never worked/unskilled mothers (37.3%) and Antigua having the highest proportion of skilled mothers (34.1%) (Table 1). Sixty-one per cent of fathers were in skilled occupations with Antiguan fathers having the highest percentage of skilled fathers (71.1%) compared with other countries (Table 1). Overall 52% of fathers lived in the home with a lower percentage of Jamaican fathers living with their child (44.3%) (Table 1). There were no significant differences by country, in birth weight, infant weight at 18 months and BMI *z*-scores at 18 months (Table 2). Twenty-one per cent of the infants were classified as ‘at risk of overweight’ and there was no significant difference in this classification by country (Table 2). Further comparisons of the parental risk factors by country comparing children classified as ‘at risk of overweight’ with those who were ‘not at risk of overweight’ are presented in online Supplementary Table S1. There were no significant differences by country.

**Table 1.** Summary of parental characteristics by country

Characteristics	Country			Total (*n* = 501)
	Jamaica (*n* = 327)	St. Lucia (*n* = 85)	Antigua (*n* = 91)	
Maternal characteristics				
** **Age [*n* (%)]*				
15–24 years	166 (50.8)	32 (38.6)	39 (42.9)	237 (47.3)
25–34 years	121 (37.0)	34 (40.9)	45 (49.5)	200 (39.9)
35–45 years	40 (12.2)	17 (20.5)	7 (7.7)	64 (12.8)
** **Education [*n* (%)]				
⩽Grade 10 of secondary school	149 (45.6)	35 (41.7)	43 (47.8)	227 (45.3)
⩾Grade 11	178 (54.4)	49 (58.3)	47 (52.2)	274 (54.7)
Occupation [*n* (%)]*				
Unemployed/unskilled	122 (37.3)	24 (28.9)	24 (26.4)	170 (33.9)
Semi-skilled	137 (41.9)	42 (50.6)	36 (39.5)	215 (42.9)
Skilled	68 (20.8)	17 (20.5)	31 (34.1)	116 (23.2)
** **Depressive symptoms score [mean (s.d.)]	16.1 (10.6)	13.1 (10.1)	12.6 (11.2)	14.9 (10.7)
** **Vocabulary score [mean (s.d.)]	146.1 (27.2)	157.9 (28.0)	162.6 (26.1)	151.0 (28.0)
Paternal characteristics				
Occupation [*n* (%)]^a^*				
Unemployed/unskilled	51 (15.6)	13 (15.5)	10 (11.1)	74 (14.8)
Semi-skilled	84 (25.7)	14 (16.7)	16 (17.8)	114 (22.7)
Skilled	177 (54.1)	54 (64.3)	64 (71.1)	295 (58.9)
Missing	15 (4.6)	3 (3.5)	0	18 (3.6)
Paternal presence [*n* (%)]*				
Does not live with the child	182 (55.7)	26 (31.3)	33 (36.3)	241 (48.1)
Lives with the child	145 (44.3)	57 (68.7)	58 (63.7)	260 (51.9)

a *n* = 483 (numbers different to follow-up sample due to missing data).

*p = <0.05 (differences by country using *t* test or χ^2^ as needed).

**Table 2.** Summary of infant characteristics by country

Characteristics	Country			Total
	Jamaica ^a^M = 52, F = 48	St. Lucia M = 47, F = 53	Antigua M = 56, F = 44	
Birth weight (kg) [mean (s.d.)]	3.2 (0.4)	3.2 (0.5)	3.1 (0.6)	3.2 (0.5)
18-month Weight (kg)	11.05 (1.4)	11.04 (1.6)	11.38 (1.5)	11.11 (1.5)
18-month BMI *z*-scores [mean (s.d.)]	0.23 (0.9)	0.03 (1.2)	0.23 (0.9)	0.19 (1.0)
18-month HAZ scores [mean (s.d.)]	−0.25 (1.0)	0.04 (1.1)	0.13 (1.2)	−0.13 (1.1)
18-month overweight categories [*n* (%)]				
Underweight/thin	6 (1.8)	3 (3.6)	1 (1.1)	10 (2.0)
Normal weight	251 (76.8)	67 (79.8)	68 (75.6)	386 (77.0)
At risk of overweight	56 (17.1)	10 (11.9)	17 (18.9)	83 (16.6)
Overweight	14 (4.3)	4 (4.7)	4 (4.4)	22 (4.4)

a M, %male; F, %female; underweight: BMI *z*-score <−2; normal weight: BMI *z*-score ≥−2 to 1; at risk for overweight: BMI *z*-score >1 to <2; overweight: BMI *z*-score >2.

Random-effects logistic regression models were developed to compare the ‘at risk of overweight’ group (OWG) and the ‘not overweight’ group (NOWG). A 1 kg difference in birth weight was associated with a fourfold increase in the odds of risk of overweight [OR (95% CI) per 1 kg difference 4.74 (2.76–8.14)]. This variable was included in all subsequent models. All models also controlled for the intervention group and family SES. Model 1 included maternal characteristics at recruitment: age, education, occupation, receptive vocabulary score, depressive symptoms and SES. Higher depressive symptoms in the mother was associated with decreased odds of risk of overweight in the sample [OR (95% CI) per 1 s.d. difference 0.75 (0.58–0.98)] (Table 3). None of the other variables included were independent predictors of overweight. Model 2 focused on the paternal characteristics at recruitment and included occupation and the presence of the father in the home along with SES of the family. Higher occupation category for the father [OR (95% CI) 0.48 (0.26–0.88)] and the presence of the father in the home was associated with decreased odds of overweight in these infants [OR (95% CI) 0.61 (0.39–0.98)] (Table 3). Model 3 included maternal and paternal variables significant in the prior models. Maternal depressive symptoms [OR (95% CI) 0.73 (0.56–0.95)], skilled paternal occupation [OR (95% CI) 0.46 (0.25–0.85)] and the presence of the father in the home [OR (95% CI) 0.60 (0.37–0.96)] were associated with decreased odds of overweight in this sample of Caribbean infants (Table 3).

**Table 3.** Random-effects logistic regression models comparing parental characteristics and odds of overweight in Caribbean infants

	Model 1			Model 2			Model 3		
Characteristics	OR	95% CI	*p* value	OR	95% CI	*p* value	OR	95% CI	*p* value
Maternal characteristics									
Age									
15–24 years	1.00	1.00		–	–		–	–	
25–34 years	0.96	0.58–1.59	0.78	–	–		–	–	
35–45 years	0.76	0.35–1.63	0.43	–	–		–	–	
Education (grade completed)	0.90	0.53–1.53	0.39	–	–		–	–	
Occupation									
Unskilled/unemployed	1.00	1.00		–	–		–	–	
Semi-skilled	1.14	0.64–2.03	0.76	–	–		–	–	
Skilled	1.50	0.77–2.93	0.30	–	–		–	–	
PPVT score (per s.d. difference)	0.99	0.98–1.00	0.14	–	–		–	–	
Depressive symptoms (per s.d. difference)	0.75*	0.58–0.98	0.03	–	–		0.73*	0.56–0.95	0.02
Birth weight	4.72	2.75–8.11	0.00	4.85	2.81–8.36	0.00	4.75	2.76–8.19	0.00
Paternal characteristics									
Occupation									
Unskilled/unemployed	–	–		1.00	1.00		1.00	1.00	
Semi-skilled	–	–		0.50	0.24–1.02	0.06	0.49	0.24–1.01	0.05
Skilled	–	–		0.48*	0.26–0.88	0.02	0.46*	0.25–0.85	0.02
Presence in home	–	–		0.61*	0.39–0.98	0.04	0.60*	0.37–0.96	0.03
Socioeconomic status (family)	0.98	0.76–1.27	0.76	1.06	0.83–0.98	0.62	0.97	0.76–1.25	0.85

*p = <0.05; all models controlling for the intervention group and infant birth weight.

PPVT, Peabody Picture Vocabulary Test, Version IV.

We also determined intraclass correlation coefficients for BMI category, birth weight and parental characteristics by country (online Supplementary Table S2). Although there was variability in the ICCs, these would not have a substantial influence on the estimates derived from our analysis.

### Qualitative

The mean age of the 30 mothers who participated in the focus group discussions was 28.3 ± 6.3 years and 63% were unemployed. The average age of the children was 14.4 ± 7.2 months with 14 girls and 16 boys. Themes that emerged from the discussions included definition of familial roles (which parent fulfils a particular role in the family, e.g. financial, nurturing and caregiving) and the importance of support (family members provide support for the caregiving of the child) related to positive health behaviours. The focus group discussions also revealed maternal perceptions of the influence of the father on the general care and feeding of young children. Several mothers believed that fathers influenced decision making especially through reinforcement of positive childcare practices and health behaviours. Despite this perception, other mothers felt unsupported and believed that the child's father was disengaged from some or all aspects of their children's lives, forcing them to make all the childcare-related decisions.

#### Familial roles

Some (9 of 30) mothers stated that fathers were not part of the decision-making process for their child. As one mother stated ‘*I take care of her by myself so he doesn't have a say right now*’ and another mother said ‘*Father is always at work he is never there, so I have to make the decisions of both the mother and the father.*’ One mother also stated ‘*They (Fathers) don't do that part, my baby father would say he doesn't know what she is supposed to eat and that is not his job.*’ For most (17 out of 30) mothers, however, the fathers positively influenced decisions on healthier meal options for infants and some were also actively involved in food preparation. Mothers stated that ‘[…] *my baby father get up in the morning and make porridge and him blend up fruit and juice*…’, and ‘[…] *him father say the snacks them a problem and he take them and give the other pickney them* (other children).’

#### Support for positive health behaviours

Family support for the mother with the caregiving of the child and promoting healthy behaviours was perceived by the mothers as important. The majority (20 of 30) mothers perceived fathers as influencing positive health behaviours when interacting with their child. Fathers were also actively encouraging mothers to breastfeed the infants as one mother stated ‘*Well my baby father every time he hears her cry I would give her from the bottle but he would always ask me why am I giving her the bottle, he wants me to breastfeed her.*’ Some (9 of 30) mothers only displayed healthy behaviours in the presence of the father as one mother stated ‘*His father behaves like he is a nutritionist so I have to give him healthy foods when he is present. I give the baby sweets and other snacks when he is not looking.*’ Another stated ‘*His father does not believe in snacks you know? I have to hide away from him when I give him snacks, he (father) always said instead of giving the child snacks you should give him an apple or even an orange.*’ These responses point to the influence of paternal support in encouraging positive health behaviours when feeding infants.

## Discussion

Our findings support the importance of parental characteristics and, in particular, fathers’ characteristics in influencing the risk for overweight in this sample of Caribbean infants. The quantitative analyses showed lower odds of risk of overweight in those infants whose fathers were present in the home after adjusting for SES, which might be expected to be higher where fathers are present. This supports the potential influence of paternal involvement in the growth of the child. Data from the focus group discussions demonstrated the perceived views of mothers, that fathers are important in promoting positive health behaviours and influencing decisions concerning nutrition for their child. Promoting a supportive environment where both parents feel engaged and can be actively involved in developing healthy behaviours in their children is a potential target for interventions to achieve healthy child growth.

Households where the mother is the only parent have been associated with a child being in a higher BMI category and being overweight, after controlling for maternal BMI, education and SES [5, 19]. Our findings support the presence of the father as a factor that may reduce the risk of overweight in young children. This association was independent of SES and paternal occupation, which suggests that the father's role outside of being the provider of the family is important for the health of the child. The association between higher paternal occupation and decreased risk of overweight in these Caribbean children may indicate that these fathers have improved health knowledge and decision-making ability because of their higher skill and education levels. For example, fathers may benefit their children's health by increased levels of physical activity, both in terms of the actual activities and encouraging their children to become more active [20].

Fathers can influence their child's caregiving environment, negatively or positively, by influencing the mother's decisions and actions and by actively participating in caregiving. Including them in interventions may provide an opportunity for them to promote positive health behaviours. Fathers who are present in the home may be able to assist in encouraging positive health behaviours even though this is not a consistent finding in the literature. A recent study utilizing data from Australian mothers points to the diversity in the roles displayed by the father where some Australian fathers were completely uninvolved and sometimes encouraged unhealthy behaviours in the home [21]. These unhealthy paternal behaviours are similar to those found in the experiences of Hispanic mothers in the USA who stated that fathers encouraged early weaning and actually fed infants snacks and sugar-sweetened beverages during early childhood [18]. However, some of the fathers described by Jamaican mothers in the focus group discussions seem to encourage healthy behaviours in their children. This suggests that fathers may be willing to be engaged in interventions to promote positive healthy behaviours in their children. This is an interesting finding which should be further explored, including focus groups targeting fathers of infants, both those who are actively and not so actively involved in the care of their children.

Maternal occupation, age, receptive vocabulary and education were not significantly associated with the risk of overweight in these infants. This may be partly explained by the mothers in the sample being predominantly low- or lower middle-income women, which may have limited the variability in some of these measures. Higher maternal depressive symptoms in this sample were associated with decreased risk of child overweight. A previous study conducted in Jamaica found that mothers of undernourished infants reported higher depressive symptoms than mothers of adequately nourished infants [22]. In a systematic review, in low- and middle-income countries, maternal depression was associated with underweight and stunting [23]. Therefore, the finding in our sample of higher maternal depressive symptoms being associated with reduced risk of overweight may be partly explained by an association between maternal depression and increased risk of child underweight. The mean depressive symptoms score for mothers of underweight children in this study was 21.6 (s.d. 3.77) in comparison with 14.9 (s.d. 10.7); for the total sample, however, there were few underweight children.

The strengths of the study include the use of mixed methodology which increased the ability to understand some of the findings of the quantitative study and improves our ability to develop interventions within the Caribbean setting. The assessment of the association of factors measured in early infancy with the risk of overweight at 18 months in a sample of children from three Caribbean countries children is another strength and the findings may be generalizable to other similar Caribbean countries. The sample size for the quantitative study was adequately powered to assess the effect of the early life exposures on the risk of overweight in these children.

The qualitative study was conducted only in Jamaica and with a sample of mothers who did not participate in the quantitative study, however we did include mothers from both an urban and rural setting. Thematic saturation may not have been achieved with this sample but most of the themes were repeated in all focus groups. Some of the baseline exposures that may have influenced the risk of overweight were not collected, so future research studies should include these such as more paternal characteristics and parent anthropometry.

Childhood overweight is a global health concern for which there is limited information on risk factors outside of high-income countries. The results of this study show the potential influence of fathers on early childhood overweight and suggest that engaging both mothers and fathers may benefit the impact of childhood overweight prevention interventions. Further research is needed to better understand the family and environmental characteristics that may influence childhood overweight in the Caribbean and other developing regions.

## Supplementary material

For supplementary material accompanying this paper visit https://doi.org/10.1017/gheg.2018.12. click here to view supplementary material
