# Supplementary material for: The role of fathers in overweight prevention: an analysis of a Caribbean cohort
Source: Glob Health Epidemiol Genom. 2018 Aug 28;3:e15. doi: 10.1017/gheg.2018.12 (PMC6152493; doi:10.1017/gheg.2018.12)
Supplement: Supplementary file 1 [file S205442001800012Xsup001.docx]

Supplementary Table 2: Comparison of children by ‘Risk of Overweight’ for all the parental characteristics by country.

| **Characteristic** | **Jamaica** | | **St. Lucia** | | **Antigua** | |
| --- | --- | --- | --- | --- | --- | --- |
|  | **Not At Risk** | **At Risk** | **Not At Risk** | **At Risk** | **Not At Risk** | **At Risk** |
| **Maternal Age (n(%))** |  |  |  |  |  |  |
| 15-24 | 128 (77.6) | 37 (22.4) | 28 (87.5) | 4 (12.5) | 33 (84.6) | 6 (15.4) |
| 25-35 | 95 (78.5) | 26 (21.5) | 30 (88.2) | 4 (11.8) | 30 (68.2) | 14 (31.8) |
| 35-45 | 34 (85.0) | 6 (15.0) | 12 (70.6) | 5 (29.4) | 6 (85.7) | 1 (14.3) |
|  |  |  |  |  |  |  |
| **Maternal Education (n(%))** |  |  |  |  |  |  |
| <Grade 10 of Secondary School | 122 (81.9) | 27 (18.1) | 29 (85.3) | 5 (14.7) | 34 (79.1) | 9 (20.9) |
| >Grade 11 | 135 (76.3) | 42 (23.7) | 41 (83.7) | 8 (16.3) | 35 (74.5) | 12 (25.5) |
|  |  |  |  |  |  |  |
| **Maternal Occupation (n(%))** |  |  |  |  |  |  |
| Unemployed/Unskilled | 97 (79.5) | 25 (20.5) | 21 (87.5) | 3 (12.5) | 19 (79.2) | 5 (20.8) |
| Semi-skilled | 110 (80.3) | 27 (19.7) | 34 (80.9) | 8 (19.1) | 29 (82.9) | 6 (17.1) |
| Skilled | 50 (74.6) | 17 (25.4) | 15 (88.2) | 2 (11.8) | 21 (67.7) | 10 (32.3) |
|  |  |  |  |  |  |  |
| **Depressive Symptoms (Mean(SD))** | 16.7 (0.68) | 13.8 (1.1) | 13.6 (1.2) | 10.2 (2.6) | 13.2 (1.3) | 11.1 (2.8) |
| **Vocabulary Score (Mean(SD))** | 145.7 (1.7) | 147.4 (3.2) | 159.1 (3.4) | 151.1 (6.5) | 162.3 (3.1) | 163.2 (6.3) |
|  |  |  |  |  |  |  |
| **Paternal Occupation (n(%))** |  |  |  |  |  |  |
| Unemployed/Unskilled | 36 (70.6) | 15 (29.4) | 10 (76.9) | 3 (23.1) | 4 (40.0) | 6 (60.0) |
| Semi-skilled | 67 (79.8) | 17 (20.2) | 11 (78.6) | 3 (21.4) | 15 (93.7) | 1 (6.3) |
| Skilled | 141 (80.1) | 35 (19.9) | 47 (87.0) | 7 (13.0) | 49 (77.8) | 14 (22.2) |
|  |  |  |  |  |  |  |
| **Paternal Presence (n(%))** |  |  |  |  |  |  |
| Does Not Live with Child | 136 (75.1) | 45 (24.9) | 22 (84.6) | 4 (15.4) | 24 (72.7) | 9 (27.3) |
| Lives with Child | 121 (83.5) | 24 (16.5) | 48 (84.2) | 9 (15.8) | 45 (78.9) | 12 (21.1) |
|  |  |  |  |  |  |  |

Supplementary Table 3: Intra-class correlation coefficients for BMI category and key exposures by country.

| **Outcomes** | **Jamaica** | **St. Lucia** | **Antigua** |
| --- | --- | --- | --- |
| BMI Category* | 0.014 | 0.077 | 0.118 |
| Maternal Education* | 0.009 | 0.000 | 0.031 |
| Vocabulary Score | 0.046 | 0.029 | 0.000 |
| Depressive Symptoms | 0.073 | 0.047 | 0.043 |
| Maternal Age* | 0.015 | 0.130 | 0.093 |
| Maternal Occupation* | 0.000 | 0.104 | 0.008 |
| Paternal Occupation* | 0.033 | 0.003 | 0.031 |
| Paternal Presence* | 0.000 | 0.069 | 0.000 |
| Birth weight | 0.091 | 0.015 | 0.102 |
|  |  |  |  |

* Variables entered as binary outcomes for the analyses
